# RNNCon: Contribution Coverage Testing for Stacked Recurrent Neural Networks

**DOI:** 10.3390/e25030520

**Published:** 2023-03-17

**Authors:** Xiaoli Du, Hongwei Zeng, Shengbo Chen, Zhou Lei

**Affiliations:** 1 School of Computer Engineering and Science, Shanghai University, Shanghai 200444, China; 2 Shanghai Key Laboratory of Computer Software Testing and Evaluating, Shanghai 201112, China

**Keywords:** coverage metrics, recurrent neural networks, software testing, deep learning

## Abstract

Recurrent Neural Networks (RNNs) are applied in safety-critical fields such as autonomous driving, aircraft collision detection, and smart credit. They are highly susceptible to input perturbations, but little research on RNN-oriented testing techniques has been conducted, leaving a threat to a large number of sequential application domains. To address these gaps, improve the test adequacy of RNNs, find more defects, and improve the performance of RNNs models and their robustness to input perturbations. We aim to propose a test coverage metric for the underlying structure of RNNs, which is used to guide the generation of test inputs to test RNNs. Although coverage metrics have been proposed for RNNs, such as the hidden state coverage in RNN-Test, they ignore the fact that the underlying structure of RNNs is still a fully connected neural network but with an additional “delayer” that records the network state at the time of data input. We use the contributions, i.e., the combination of the outputs of neurons and the weights they emit, as the minimum computational unit of RNNs to explore the finer-grained logical structure inside the recurrent cells. Compared to existing coverage metrics, our research covers the decision mechanism of RNNs in more detail and is more likely to generate more adversarial samples and discover more flaws in the model. In this paper, we redefine the contribution coverage metric applicable to Stacked LSTMs and Stacked GRUs by considering the joint effect of neurons and weights in the underlying structure of the neural network. We propose a new coverage metric, RNNCon, which can be used to guide the generation of adversarial test inputs. And we design and implement a test framework prototype RNNCon-Test. 2 datasets, 4 LSTM models, and 4 GRU models are used to verify the effectiveness of RNNCon-Test. Compared to the current state-of-the-art study RNN-Test, RNNCon can cover a deeper decision logic of RNNs. RNNCon-Test is not only effective in identifying defects in Deep Learning (DL) systems but also in improving the performance of the model if the adversarial inputs generated by RNNCon-Test are filtered and added to the training set to retrain the model. In the case where the accuracy of the model is already high, RNNCon-Test is still able to improve the accuracy of the model by up to 0.45%.

## 1. Introduction

In recent years, the improvement in the computing power of computer hardware and the development of artificial intelligence techniques have made it possible to create larger and deeper Deep Neural Networks(DNNs) in a shorter time. Deep learning techniques have made tremendous breakthroughs in Natural Language Processing [1,2], Computer Vision [3], and Automatic Speech Recognition [4]. Despite the impressive capabilities of DL systems, they can exhibit misbehavior due to data bias, overfitting, underfitting, etc. [5]. These misbehaviors can lead to catastrophic results in safety-critical areas. For example, misdiagnosis in healthcare distorts health assessment; car crashes due to incorrect predictions of self-driving cars, and so on. As DL systems are deployed in more and more areas critical to personal safety, property safety, and even national security, such as facial recognition, autonomous driving [6], smart credit and aircraft collision detection, the Blue Book of “Security Framework for Artificial Intelligence” released by the Security Institute of the China Academy of Communications in December 2020 clearly states that it is imminent to improve the robustness [7], correctness [8], security, and interpretability [9,10,11] of DNNs.

Software testing is an important way to ensure software quality. Different from traditional software systems, DL systems essentially follow a data-driven programming paradigm, and their decision logic is derived from training data and evolves over time in response to frequently provided new data [12]. Automated testing and systematic testing of large-scale, real-world DL systems with thousands of neurons and millions of parameters are extremely challenging issues [13].

The general approach to testing DL systems is to collect and manually label as much real-world test data as possible. Google’s DL system for self-driving cars generates synthetic training data through simulations. However, such simulations do not take into account the internal decision logic of the models, making it difficult to find data or tests that can make the model’s predictions wrong.

For the large input space of real-world DL systems, such as all possible road conditions faced by self-driving cars, the above methods are not effective enough at finding corner cases.

In order to find the corner cases of DNNs, Pei et al. [13], Ma et al. [14,15], Sun et al. [16,17,18], Zhou et al. [19], Xie et al. [20], Tian et al. [21] and Guo et al. [22], etc., have conducted a great amount of research.

Both CNNs and RNNs are important components of DNNs, but current research has focused on CNNs [23] that perform well in image processing tasks, and little research has been done on RNNs. RNNs benefit from its recurrent cell and perform well in tasks dealing with sequential data, and are widely used in Language Model [24], Image Processing [25,26], Sentiment Analysis [27], etc.

RNNs are susceptible to input perturbations and have quality problems. Since the structure of CNNs is different from that of RNNs, testing techniques oriented to CNNs are difficult to be applied accurately to RNNs. Current testing techniques for RNNs are limited, leaving a threat to a large number of sequential application domains. To bridge such a gap and improve the generalization performance and robustness of RNNs, custom coverage criteria such as Hidden State Coverage (HS_C) [28], Boundary Coverage (BC), Stepwise Coverage (SC), and Temporal Coverage (TC) [29] have been proposed to guide the testing by Guo et al. [28], Du et al. [30], Huang et al. [29]. However, what these researchers have not considered is that the underlying structure of RNNs is still a fully connected neural network with an additional virtual unit “delayer” to record the network state at the time of data input, and we can still use the neurons and weights as the minimum unit of RNNs. If we use the recurrent cell as the minimum unit for testing, it is easy to miss the finer-grained logical structure inside the recurrent cell, resulting in the defects of the RNNs model not being adequately found. A hidden state is the output of a recurrent cell, which is a vector of hundreds or thousands of units. We note that neurons are ambiguous in RNNs, and in line with Huang et al. [29], we consider that each element in the hidden state vector can be considered as a neuron. However, this does not mean that coverage testing techniques for CNNs can be directly applied to RNNs. Considering the different network structures, coverage testing techniques for RNNs need to be redefined for the particular structure of RNNs. Inspired by Zhou et al.’s study [19], we use the computational graph unfolding technique [31] to unfold the RNNs at a given input to extract contributions. Contribution is a term that refers to the combination of the output of a neuron with the weights it emits. The contribution coverage metric proposed in the work of Zhou et al. is for CNNs. In the convolutional layer, the elements in the filter are treated as neurons [32]. It is not possible to apply this contribution coverage metric directly to RNNs with recurrent structure, both in definition and in terms of concrete implementation.

**Figure 1:**
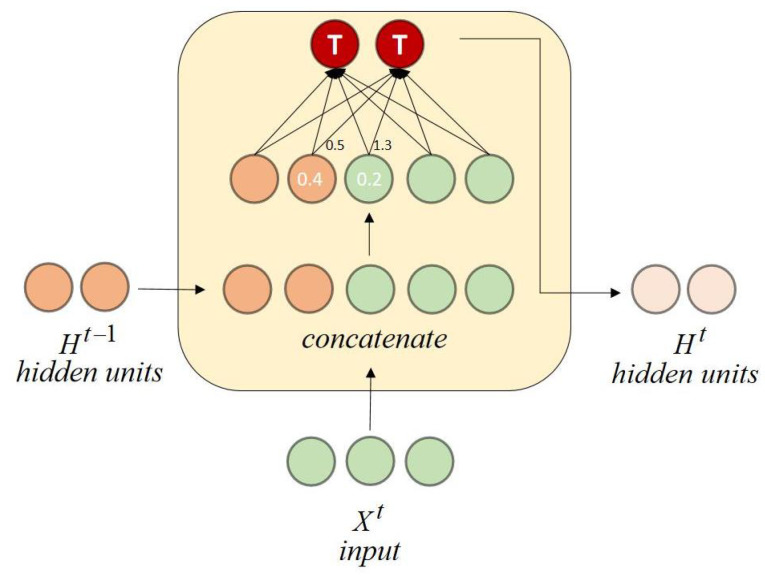
Vanilla RNN cell. The numbers in nodes and the numbers on edges represent the outputs of the neurons and the weights, respectively. The text T in the nodes is the sum of the weights of neurons from the previous layer, and is activated by a tanh function.

It is well known that connections emitted by different neurons have different weights, and the contribution of a neuron with a large output but a small weight may be smaller than that of another neuron with a small output but a large weight [19]. For example, a vanilla RNN cell refers to the simplest RNN architecture, and we use an input size of 3 (green) and 2 hidden units (orange) with a batch size of 1, as shown in Figure 1. The numbers in nodes and the numbers on edges represent the outputs of neurons and weights, respectively. For ease of presentation, we denote neurons with output 0.4 and 0.2 as node1 and node2, respectively. Because 0.4×0.5<0.2×1.3, so even if node1>node2, node2 contributes more to the prediction results than node1.

Therefore, we believe that it is difficult to reveal the correct decision mechanism of RNNs by considering only the hidden states of RNNs or by considering only neurons and ignoring weights, and it is may even more difficult to accurately find effective corner cases of RNNs.

Stacked RNNs are comprised of multiple hidden RNN layers where each layer contains multiple memory cells. Stacking RNN hidden layers makes the model deeper and more accurately earns a description as a DL technique. The depth of neural networks usually leads to the success of the method on a wide range of challenging prediction problems [33,34]. Different from existing metrics, we want to know the possible reasons for the success of Stacked RNNs.

In this paper, we propose a scalable contribution coverage RNNCon for the underlying structures of Stacked RNNs. We redefine contributions in RNNs, formalize the process of contribution extraction, and also define how to calculate contribution coverage rate to reflect the diversity of test inputs. When a contribution is larger than the threshold we specified, it indicates that the contribution is activated. We form a corresponding test framework prototype RNNCon-Test, which outperforms RNN-Test [28]. Unlike the study by Zhou et al., we do not generate perturbations superimposed on the test inputs by jointly maximizing the contributions and the outputs of the contribution-connected neurons. We simply take an inactivated contributions as a loss and maximize the loss by a gradient ascent technique to generate the perturbation. The generated perturbations are more mild and imperceptible compared to those generated in the work of Zhou et al. They can be superimposed on the input data to generate test inputs, which may not exist in the real world. So we filter out them close to the real world by measuring the naturalness of them. For supervised tasks, RNNCon-test automatically takes the labels of the seeds as the labels of the generated test inputs and feeds these labeled test inputs to the model under test. If the prediction result of a test input is inconsistent with its label, then we consider that a defect is found, and such a test input is an adversarial sample, which is consistent with the study by Harel-Canada et al. [35]. These labeled generated test inputs can also be added to the training dataset and the model can then be retrained to improve its performance and robustness. For example, RNNCon-Test can also improve the accuracy by up to 0.45% when the accuracy of the LSTM model has already reached a high level.

We validate the effectiveness and scalability of RNNCon-Test with 2 datasets, 4 LSTM models and 4 GRU models.

In summary, we make the following contributions:

- We first explicitly use finer neurons and weights within Stacked RNNs and propose a special contribution coverage metric RNNCon for Stacked RNNs by combining the outputs of neurons and the connection weights they emit. We further define the calculation method of contribution coverage rate, which reflects the diversity of test inputs. Additionally, we visualize the coverage changes at each time step of each gate to analyze the memory of GRUs [36] or LSTMs [37,38].
- We build an optimization function for inactivated contributions, and generate test inputs by gradient ascent. We adopt Inception Score(IS) [39], Fr*è*chet Inception Distance(FID) [40] and L1 distance to filter natural and valid test inputs to make them more similar to those exist in the real world. Most of the generated test inputs can make the predictions of the corresponding models wrong.
- We design and implement a test framework prototype RNNCon-Test which outperforms RNN-test [28]. It is not only effective in detecting defects and improving the performance of Stacked RNNs but can also be extended to variants of RNNs, i.e., LSTMs and GRUs.

## 2. Background

In this section, we introduce Stacked RNNs and the limitations of existing test coverage metrics.

### 2.1. Stacked Recurrent Neural Network

**Figure 2:**
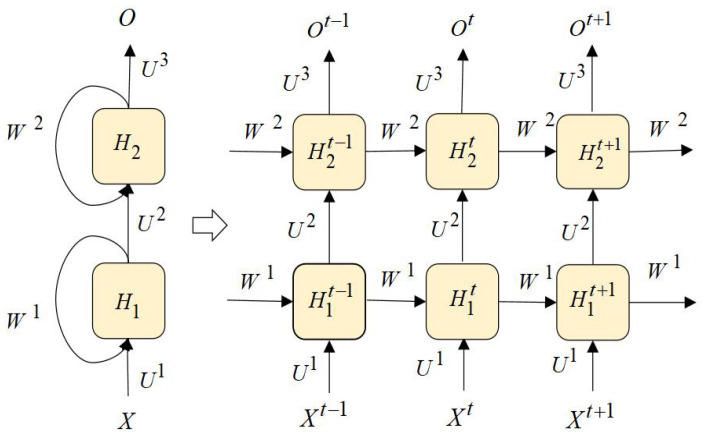
A Stacked RNN with two hidden layers between time step t−1 and t+1.

RNNs [41] take sequence data as input, recursively along the direction of sequence evolution, and all recurrent units are connected in the form of chains. A Stacked RNN containing two hidden layers between time step t−1 and t+1 is shown in Figure 2.

**Figure 3:**
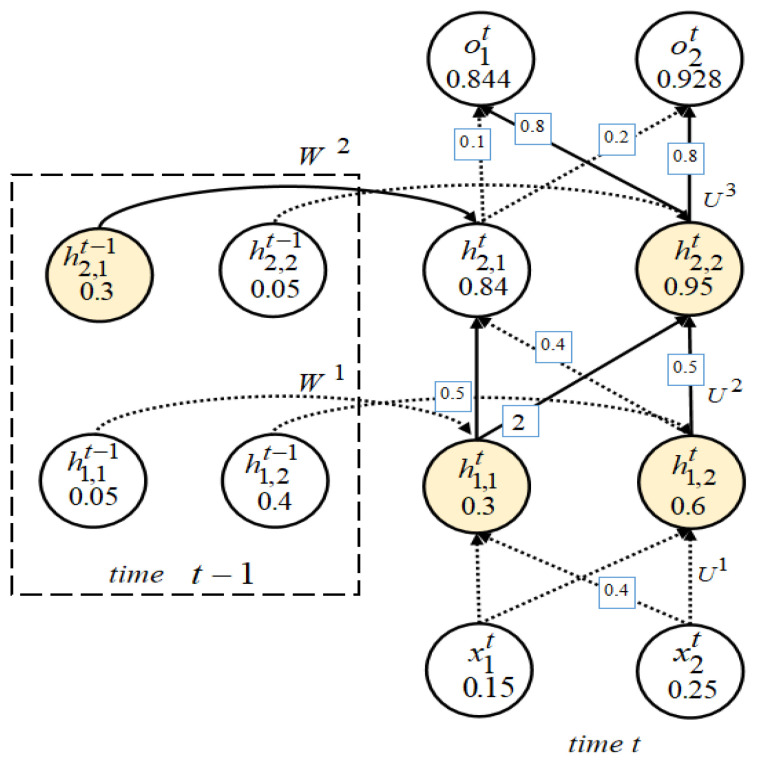
The internal structure of a Stacked RNN with the weights visible at time step *t*, allow us to observe the direction of information transfer and the importance of weights in the process of information transfer process. For weights without specified values, the default value is 1.

$X^{t}$ is the input vector at time step *t*, $O^{t}$ is the output vector at time step *t*, and $H_{1}^{t}$ and $H_{2}^{t}$ are the output vectors of the first hidden layer and the second hidden layer at time step *t*, respectively. $U^{1},U^{2},U^{3}$ are the weight matrices between each adjacent layer. $W^{1}$, $W^{2}$ are the weight connections of the first hidden layer and the second hidden layer from the previous time step to the next step, respectively. For a Stacked RNN, the value of a hidden state depends not only on the current input but also on the output of the hidden state from the previous time step. If we ignore the output of the hidden state from the previous time step, the Stacked RNN can be regarded as a simple fully connected network. We represent more fine-grained internal structure of a Stacked RNN with two hidden RNN layers at time *t*, as shown in Figure 3.

The input vector at time step *t* is denoted as $X^{t}\left(x_{1}^{t},x_{2}^{t}\right)$, the output vector is denoted as $O_{t}\left(o_{1}^{t},o_{2}^{t}\right)$, and the output vectors of the first and second hidden layers are $H_{1}^{t}\left(h_{1,1}^{t},h_{1,2}^{t}\right)$ and $H_{2}^{t}\left(h_{2,1}^{t},h_{2,2}^{t}\right)$, respectively. We can observe that if we only cover the hidden state *H* of Stacked RNNswith more than one hidden RNN layer, we will ignore a lot of fine-grained logical information, such as the combination of neuron outputs and weights and gate units. The internal processing of Stacked RNNs at each time step is still a black box to us.

For convenience, we formalize a Stacked RNN as follows.

We let *l* be the number of layers of a Stacked RNN, $s_{i}$ be the number of neurons in the *i*th layer, *e* be the total number of time steps, $n_{i,j}^{t}$ represents the *j*th neuron in the *i*th layer at the time step *t*, $N_{i}^{t}=\left\{n_{i,j}^{t}|1\leq j\leq s_{i}\right\}$. We use $u_{k,j}^{i,t}$ to denote the connection weight between input neuron $n_{i-1,k}^{t}$ and $n_{i,j}^{t}$ at the time step *t*, $U^{i,t}=\left\{u_{k,j}^{i,t}|1\leq k\leq s_{i-1},1\leq j\leq s_{i}\right\}$. Use $\omega_{j}^{i,t}$ to denote the connection weight between hidden neuron $n_{i,j}^{t-1}$ and $n_{i,j}^{t}$ from time step t−1 to *t*, $W^{i,t}=\left\{\omega_{j}^{i,t}|1\leq j\leq s_{i}\right\}$. We use a tuple $G=(N_{i}^{t},U^{i,t},W^{i,t})$ to represent a Stacked RNN. For example, $n_{1,2}^{1}$ represents the 2nd neuron in the 1st layer at time step 1. $u_{1,2}^{2,3}$ represents the connection weight between neuron $n_{1,1}^{3}$ and $n_{2,2}^{3}$ at time step 3; $\omega_{3}^{2,2}$ represents the connection weight of the 3rd neuron $n_{2,3}^{1}$ and $n_{2,3}^{2}$ in the 2nd layer between the time step 1 and the time step 2. It is worth noting that the neurons here are the same before and after, the difference is that the information processed at different time steps is different.

A neuron is the basic computational unit of a Stacked RNN. Let $h_{i,j}^{t}$ be the output of the *j*th hidden recurrent unit in layer *i* at time step *t*. $H_{i}^{t}=\left\{h_{i,j}^{t}|1\leq j\leq s_{i}\right\}$, $o_{j}^{t}$ be the output of the *j*th neuron in the output layer, $O^{t}=\left\{o_{j}^{t}|0\leq j\leq s_{l}\right\}$. The calculation of $H_{i}^{t}$ and $O^{t}$ can be viewed in Formulas (1) and (2), where *f* and *g* are both activation functions. It should be noted that although in each time step of a Stacked RNN, $U^{i,t}$ and $W^{i,t}$ are the same, the values of the combination of neuron outputs and the weights are different due to the outputs of neurons are different at each time step.

(1) $$H_{i}^{t}=f\left(U^{i,t}\cdot X^{t}+W^{i,t}\cdot H_{i}^{t-1}\right)\;$$

(2) $$O^{t}=g\left(U^{l,t}\cdot H_{l-1}^{t}\right)$$

### 2.2. Limitations of Existing Coverage Metrics

In order to evaluate the quality of RNN systems and identify the defects of the RNN systems, several studies have proposed several RNN test coverage metrics. Guo et al. [28] proposed the HS_C for RNNs and the Cell State Coverage (CS_C) criterion for LSTMs. A vanilla RNN becomes an LSTM network if we replace the recurrent units of hidden layers in vanilla RNN with LSTM units. The hidden state is the “memory” of the RNN, which is the output of the hidden layer in the previous time step. HS_C is defined as the number of hidden state outputs that reach the maximum of all candidate states and the total number of hidden states during the testing process. CS_C is similar to K-multisection neuron coverage from Ma et al.’s research [14], dividing the value range [−1,1] of the cell state in LSTM into multiple modules, if the state component of a certain unit belongs to a certain module, it means that the state component is covered by this module. These two coverage metrics only consider the coverage of one single time step, without considering the impact of the outputs from the previous time step. Such coverage metrics seem to be discrete and lack sufficient persuasiveness for testing.

Huang et al. [29] proposed BC, SC, and TC. BC sets the threshold interval of the output result of a neural network at a certain time step. If the output is outside this interval, the input can be viewed as a test, and a new test can be selected by optimizing a loss function. SC measures the degree of change in short-term memory, and TC measures the overall temporal semantics. We know that for an RNN layer, the output of a decision neuron is jointly determined by the outputs of the previous neurons and the weights they emit, as well as the retained hidden states. These coverage metrics ignore the effect of weights and do not better find flaws in the model.

## 3. Contribution Coverage of Stacked RNNs

We define the contribution in this section, and show how to extract the contribution from a trained Stacked RNN and how to calculate the contribution coverage rate.

### 3.1. Definition of Contribution in Stacked RNNs

Contribution coverage can be divided into two categories due to the “memory” function of Stacked RNNs. One is at a single time step, where the contribution is a general definition, that is, the combination of the output of a neuron and the weight it emits to connect the neurons in the next layer. The other is on multiple consecutive time steps, where the value of a hidden state depends not only on the input of the current time step but also on the output of the hidden state from previous time step. Contribution in this case is defined as the combination of the output of a neuron from the previous time step and the weight it emits to connect the same neuron of the next time step.

**Figure 4:**
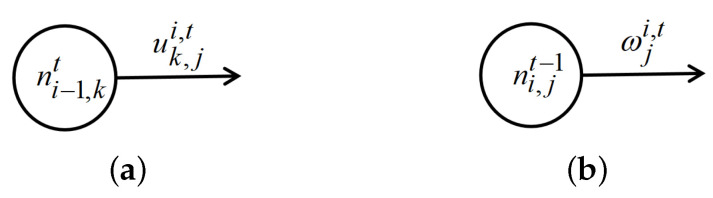
Two definitions of contributions in a stacked RNN. (**a**) A contribution at time step *t*. (**b**) A contribution between time step *t* and t+1.

**Definition** **1** (Contribution of RNN). *At a single time step, we use $cu_{k,j}^{i,t}=\left(n_{i-1,k}^{t},u_{k,j}^{i,t}\right)$ to denote the contribution between neuron $n_{i-1,k}^{t}$ and $n_{i,j}^{t}$, as shown in Figure 4a, use $c\omega_{j}^{i,t}=\left(n_{i,j}^{t-1},\omega_{j}^{i,t}\right)$ to denote the contribution between hidden neuron $n_{i,j}^{t-1}$ and $n_{i,j}^{t}$ from step t−1 to t, as shown in Figure 4b.*

### 3.2. Contribution Extraction of Stacked RNNs

In a Stacked RNN, if an input is *x* at time *t*, we use $Cu_{k,j}^{1,t}\left(x\right)$ to represent the value of contribution $cu_{k,j}^{1,t}$, $Cu_{k,j}^{i,t}\left(h_{j}^{i-1,t}\right)$ to represent the value of contribution $cu_{k,j}^{i,t}$, $C\omega_{j}^{i,t}$ to respresent the value of contribution $c\omega_{j}^{i,t}$, where $Cu_{k,j}^{1,t}\left(x\right)=u_{k,j}^{1,t}\cdot x$, $Cu_{k,j}^{i,t}\left(h_{j}^{i-1,t}\right)=u_{k,j}^{i,t}\cdot h_{j}^{i-1,t}$, $C\omega_{j}^{i,t}=\omega_{j}^{i,t}\cdot h_{j}^{i,t-1}$, $C\left(x\right)=\{Cu_{k,j}^{1,t}\left(x\right)\cup Cu_{k,j}^{i,t}\left(h_{j}^{i-1,t}\right)\cup C\omega_{j}^{i,t}|2\leq i\leq l,1\leq k\leq s_{i-1},1\leq j\leq s_{i}\}$. We normalize $Cu_{k,j}^{1,t}\left(x\right)$, $Cu_{k,j}^{i,t}\left(h_{j}^{i-1,t}\right)$ and $C\omega_{j}^{i,t}$ to between 0 and 1 for easy comparison with the threshold we specified. We use $nCu_{k,j}^{i,t}\left(h_{j}^{i-1,t}\right)$ to denote the normalized Contribution of $Cu_{k,j}^{i,t}\left(h_{j}^{i-1,t}\right)$, and use Equation (3) to normalize $Cu_{k,j}^{i,t}\left(h_{j}^{i-1,t}\right)$.

(3) $$nCu_{k,j}^{i,t}\left(h_{j}^{i-1,t}\right)=\frac{Cu_{k,j}^{i,t}\left(h_{j}^{i-1,t}\right)-min\left(C\left(x\right)\right)}{max(C(x))-min(C(x))}$$

$Cu_{k,j}^{1,t}\left(x\right)$, $C\omega_{j}^{i,t}$ are normalized in the same way as $Cu_{k,j}^{i,t}\left(h_{j}^{i-1,t}\right)$. Given an input *x*, if a contribution normalized is greater than a threshold thred, we can say that the contribution is activated by input *x*.

In Algorithm 1, for a given input *X*, the threshold thred and the model *M*. If the input *X* is an image, then $X^{t}$ is each column of pixels in the image, and if it is a text, then $X^{t}$ is a word. First, we get the output $H_{i}^{t}$ and the weight $U^{i,t}$, $W^{i,t}$ of each layer from time step *t* to image size or embedding vector length (line 1–4). In a Stacked RNN, the value of contribution ${\tilde{C}}^{1,t}$ of the input layer is expressed as the dot product of the input $X^{t}$ and the weight $U^{1,t}$ of the first layer.

**Algorithm** **1:** ConExt(X,thred,M) /*Stacked RNN Contribution extract algorithm*/

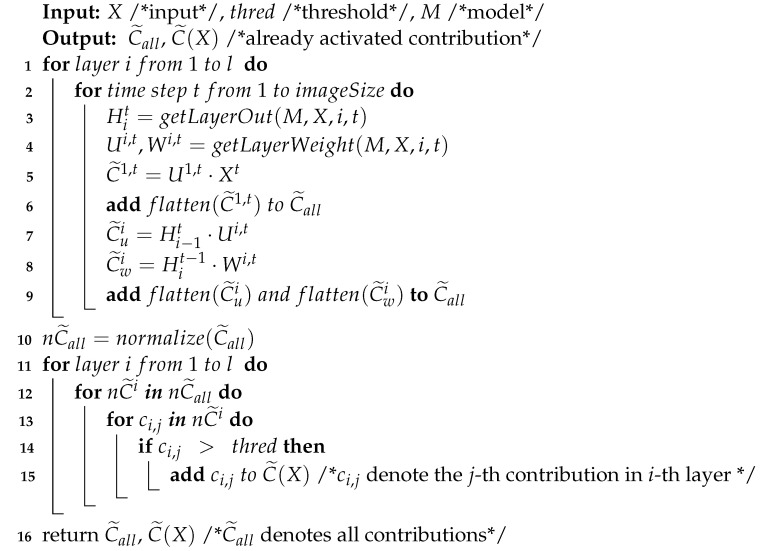



    We add the obtained values of contribution ${\tilde{C}}^{1,t}$ to ${\tilde{C}}_{all}$ for temporary storage (line 5–6). For layers other than the input layer from 2 to *l*, ${\tilde{C}}_{u}^{i}$ is equal to the dot product of $H_{i-1}^{t}$ and $U^{i,t}$, and ${\tilde{C}}_{w}^{i}$ is equal to the dot product of $H_{i}^{t-1}$ and $W^{i,t}$. flatten is a function that converts the contribution matrix into a one-dimensional vector, which helps us to extract each contribution individually (line 7–9). After the acquired contributions are normalized, we traverse them. If a contribution is greater than thred during the traverse, it means that this contribution is activated; then, add it to $\tilde{C}\left(X\right)$ (line10–15).

### 3.3. Contribution Coverage Rate of Stacked RNNs

Contribution coverage refers to the ratio of activated contributions to all contributions in the Stacked RNN. $\tilde{C}\left(X\right)$ is a set of activated contributions, and ${\tilde{C}}_{all}$ is a set of all contributions.

For input *X*, the contribution coverage of a Stacked RNN can be described in the following form.

(4) $$RConC=\frac{|\tilde{C}\left(X\right)|}{|{\tilde{C}}_{all}|}$$

Take the Stacked RNN in Figure 3 as an example. For input *X* and threshold 0.3, the number of contributions is 16. It can be observed that $Cu_{1,1}^{2,t}$, $Cu_{1,2}^{2,t}$, $Cu_{2,2}^{2,t}$, $C\omega_{1}^{2,t}$, $Cu_{2,1}^{3,t}$, $C\omega_{1}^{2,t}$, $Cu_{2,2}^{3,t}$ greater than 0.3. So at time step *t*, there are a total of 6 activated contributions; then, the contribution coverage rate of the RNN is 37.50%. The combination of the yellow circles and the solid line them emit in Figure 3 represents the contribution activated.

We compare with neuron coverage; assuming that the threshold is also 0.3, then at time step *t*, the total number of neurons is 12.

There are a total of 8 neuron outputs greater than 0.3, including $h_{1,1}^{t}$, $h_{1,2}^{t}$, $h_{2,2}^{t}$, $h_{2,1}^{t}$, $o_{1}^{t}$, $o_{2}^{t}$ at time step *t* and $h_{2,1}^{t-1}$, $h_{1,2}^{t-1}$ at time t−1. Then the coverage rate of the neuron is 66.67%.

## 4. RNNCon-Test Design

**Figure 5:**
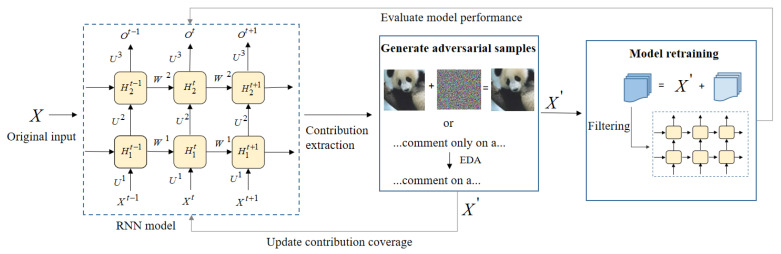
Architecture of RNNCon-Test.

RNNCon-Test mainly consists of test input generation guided by RNNCon, finding the adversarial samples, adding the adversarial samples to the training set, filtering the real and natural dataset by L1-distance, FID and IS to retrain the model, and then evaluating the performance of the retrained model, while the generated adversarial samples can be input to the tested model to update the contribution coverage. The architecture of RNNCon-Test, as shown in Figure 5. Algorithm 2 shows how test inputs are generated.

**Algorithm** **2:** GenInputs(seeds) /*coverage guided test input generation in Stacked RNN*/

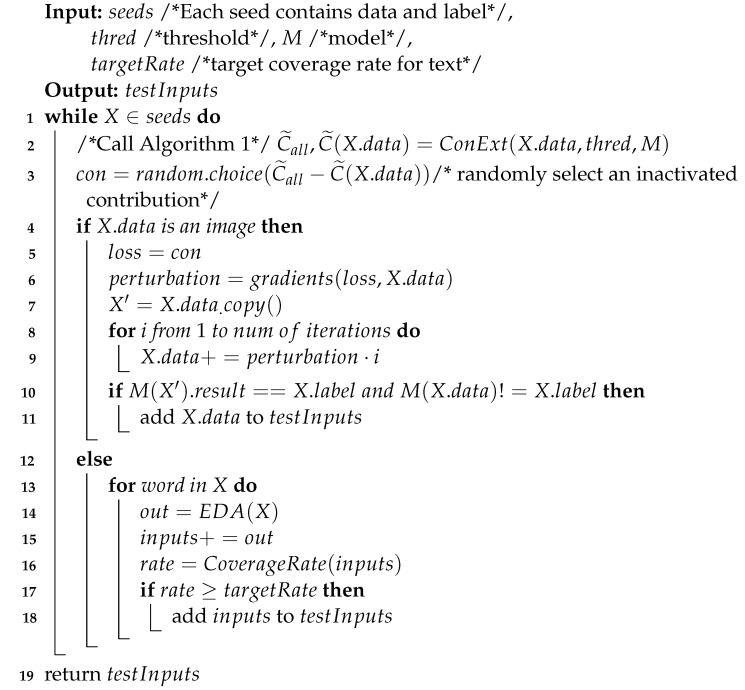



Given a set of seed inputs seeds, we call ConExt to get all the extracted contribution set ${\tilde{C}}_{all}$, and the activated contribution set $\tilde{C}\left(X\right)$. The inactivated contribution set can be easily obtained by calculating the difference between ${\tilde{C}}_{all}$ and $\tilde{C}\left(X\right)$. We randomly pick a contribution that we want to activate from the inactivated contributions (line 1–3). Activating a contribution means that the larger the value of this contribution, the better. If input *X* is an image, then each column of the image is an input for each time step. We regard the inactivated contribution as a loss, maximize the loss by gradient ascent, and superimpose the obtained perturbation on the original image to obtain new test input (line 6–9). We judge that in the model *M*, the generated test input can be considered as an adversarial sample if the prediction result of the original image is the same as its label and the prediction result of the generated image is different from the label of the original image. We add such an adversarial sample to the testInputs (line 10–11).

If input *X* is a text, then each word in the text represents an input at each time step and we cannot activate the randomly selected contribution, whereas can process the input *X* by using four operations in the Easy Data Augmentation (EDA) [42] techniques to achieve our specified coverage (line 13–18).

These four operations implement synonym replacement: randomly extract *n* words from the sentence excluding stop words, randomly find synonyms of these words, and then replace the original words with a synonym; Random insertion: randomly select a word from the sentencethat does not include stop words, and then randomly select a synonym of this word, then insert it at a random position in the original sentence; Random swap: in a sentence, randomly swap the positions of two words, repeating this process *n* times; Random deletion: every word in the sentence has probability *P* to be deleted. For the generated text inputs that can reach the coverage rate specified by us, we add it to testInputs (line 18).

## 5. Experiments

We use tensorflow-gpu2.3.0 to implement RNNCon-Test. The computer hardware configuration for all our experiments is Intel(R) Core(TM) i7-8750H CPU @ 2.20 GHz, 24G RAM and 6G NVIDIA GTX 1060 GPU.

### 5.1. Experimental Setup

MNIST [43] is a dataset containing 60,000 samples in the training dataset and 10,000 samples in the test dataset, each of which is a grayscale image of handwritten digits of size 28 × 28. MINST is used to train a five-layer LSTM model Stacked-MNIST-LSTM, a four-layer LSTM model MNIST-LSTM, a five-layer GRU model Stacked-MNIST-GRU and a four-layer GRU model MNIST-GRU. Both LSTM and GRU are variants of RNN. Gates that control the addition and forgetting of information do not affect our extraction of contributions. Except for the input layer, the first and second layers of Stacked MNIST-LSTM are both LSTM layers to extract features, and the latter two layers are fully connected layers. The same training procedure was run more than 10 times with 10 epochs each on the MNIST test dataset The average accuracy of Stacked MNIST-LSTM is 99.01%, with 3754 neurons and 58,520 contributions extracted.

Unlike stacked MNIST-LSTM, MNIST-LSTM has only one LSTM layer and its average accuracy is 98.76%, with 170 neurons and 29,848 contributions.

The GRU model is structurally similar to LSTM, except that the LSTM layer is replaced by the GRU layer. The average accuracy of Stacked-MNIST-GRU on MNIST test set is 98.90%, with 3754 neurons and 44,184 contributions extracted. The average accuracy of MNIST-GRU is 98.53%, with 170 neurons and 22,680 contributions extracted.

IMDB [44] is a dataset integrated in Keras that contains 50,000 heavily polarized reviews from the internet. Of these, 25,000 reviews are used for training and 25,000 are used for testing. Both the training set and the test set contain 50% positive reviews and 50% negative reviews.

IMDB is used to train a five-layer LSTM model Stacked-IMDB-LSTM with an embedding layer, 2 LSTM layers, and 2 fully connected layers, and another GRU model Stacked-IMDB-GRU that replaces the LSTM layers with GRU layers. The average accuracy of the Stacked-IMDB-LSTM on the test set is 84.68%, and there are 64,161 neurons and 66,592 contributions extracted. The accuracy of the Stacked-IMDB-GRU on the test set is 86.21%, with 64,161 neurons and 50,208 contributions extracted.

To compare who can better show the internal decision mechanism of the model in terms of neurons and contributions, we analyzed the number of neurons and contributions for RNNs with the same number of network layers but different recurrent units, e.g., MNIST-LSTM and MNIST-GRU, in Table 1. We can find that the number of neurons in the GRU model and LSTM model is the same, but the number of contributions of the GRU model is significantly less than that of the LSTM model, which confirms that the design of GRU is indeed simpler than that of LSTM. It also shows that contribution coverage can better represent the logic inside the model than neuron coverage. Details of the models and datasets we used are shown in Table 1, where #Cons is the size of ${\tilde{C}}_{all}$ after flattening.

We investigated the following questions.

RQ1: How strong is the coverage capacity of RNNCon?

RQ2: How is the effectiveness of the RNNCon-Test?

RQ3: How is the quality of the adversarial inputs generated by RNNCon-Test? Can retraining improve the performance of the model?

We first evaluate the coverage capacity of RNNCon and its adaptability on different models. We also evaluate the ability of RNNCon-Test to activate inactivated contributions in image and text tasks. Then, we evaluate the quality of the test input generated by RNNCon-Test and compared it with RNN-Test [28]. Finally, we evaluate how much the performance of the model couldcan be improved if the model is retrained with adversarial inputs added to the training data.

### 5.2. Coverage Results (RQ1)

In this section, we evaluate the coverage capacity of RNNCon on LSTMs and GRUs.

**Figure 6:**
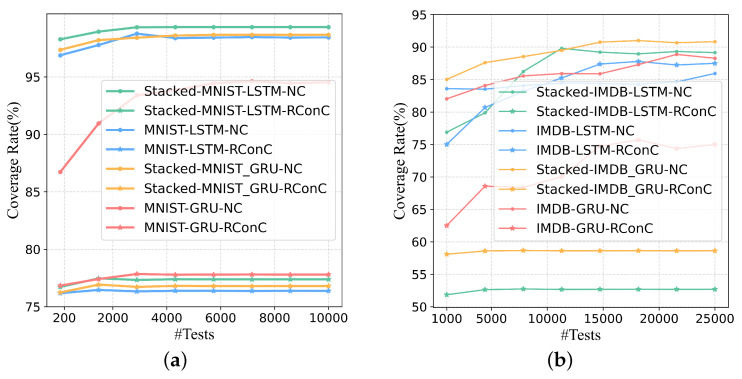
The growth trend of coverage of each model when increasing test inputs. RConC and NC are the values of RNNCon coverage and Neuron coverage respectively. The threshold is 0.25 for MNIST and 0.5 for IMDB. (**a**) MNIST. (**b**) IMDB.

When increasing test inputs, the growth trend of RConC and NC of each model is shown in Figure 6. Note that the coverage here represents the coverage of the LSTM layer or GRU layer. For Stacked MNIST-LSTM and Stacked MNIST-GRU, we calculate the coverage of the LSTM and GRU layers of the first layer.

First, in each model trained with MNIST and IMDB datasets, the RConC > NC is maintained only when the number of tests fed to the IMDB-LSTM is greater than 15,000. Other models keep RConC < NC as the number of tests increases. Therefore, for the eight models in Table 1, RNNCon has stronger coverage capability than neuron coverage., because RNNCon covers fewer internal logical parts of the model under the same threshold. If there are more uncovered logic parts, more selectable inactived neurons, and more searchable space for generating adversarial samples, then it is more likely that more defects will be found in the corresponding model. We suggest using RNNCon metric to guide the generation of test inputs rather than using it alone. Consistent with Huang et al.’s point of view, RNNCon does not have to be closely related to adversarial samples [45,46]. Here, the number of successfully generated adversarial samples is defined as the number of model defects found, which is consistent with Harel-Canada et al.’s opinion [35].

Second, with the increase of the number of tests, both RConC and NC increase more slowly and finally become stable. More test inputs with different features can activate more contributions or neurons. When the test inputs reach a certain scale, their feature distribution has been relatively determined, and the coverage tends to be stable. Even with enough data, some contributions are hard to be activated. However, once these hard-to-activate contributions are activated by the test inputs we generate, then such test inputs are likely to affect the model’s prediction results, and these contributions become the attack points of the model.

Third, for the MNIST-trained model, the reason why we choose the threshold value of 0.25 is that when the threshold value is equal to 0.25, the maximum difference of RConC between different labels (0–9) is greater than the threshold value of 0 and 0.5. At this time, RConC can better distinguish different features and better show the internal mechanism of the corresponding model. The above method is also used for threshold selection of IMDB training model. In general, for single-layer LSTM or GRU layers, RNNCon can cover deeper internal decision mechanisms of RNNs with stronger coverage capacity than neuron coverage.

**Figure 7:**
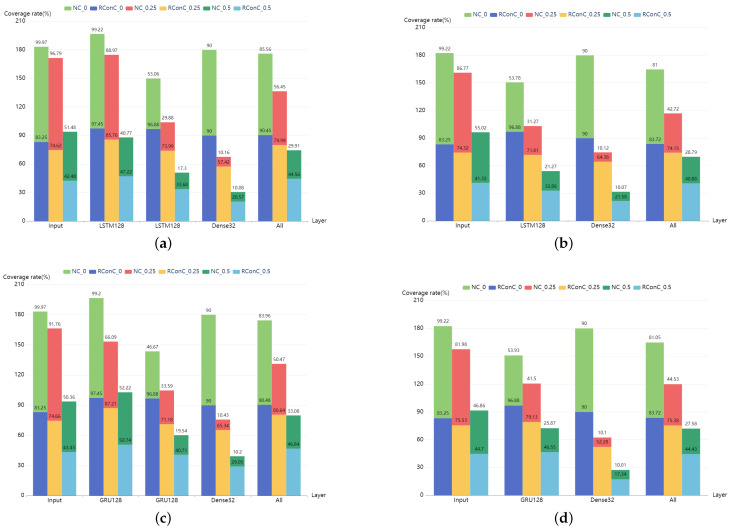
The RConC of each layer of MNIST model is compared with NC under different thresholds of 0, 0.25, and 0.5 for one same test input. 0 in NC_0 indicates that the threshold we specify is 0. LSTM128 indicates that this layer is an LSTM layer with 128 LSTM units. All indicates the coverage of the entire model, including all layers except the output layer. The number at the top of the column is the coverage rate of the corresponding layer. (**a**) Stacked-MNIST-LSTM. (**b**) MNIST-LSTM. (**c**) Stacked-MNIST-GRU. (**d**) MNIST-GRU.

**Figure 8:**
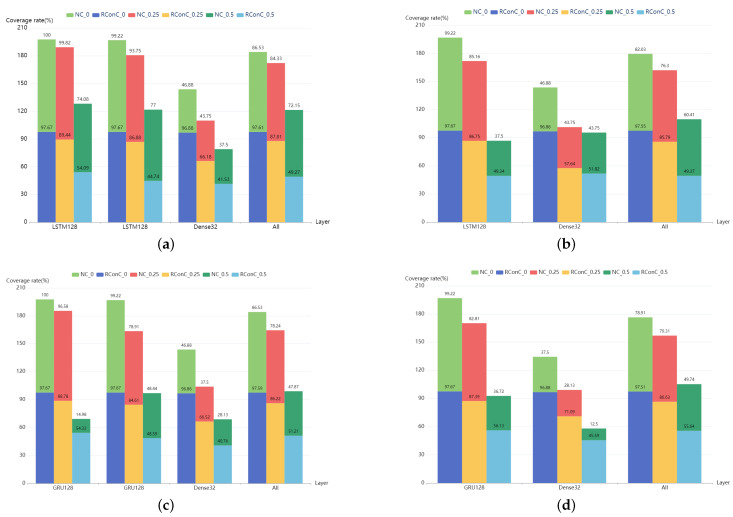
The RConC of each layer of IMDB model is compared with NC under different thresholds of 0, 0.25, and 0.5 for one same test input. The meanings of other parameters are the same as those in Figure 4. (**a**) Stacked-IMDB-LSTM. (**b**) IMDB-LSTM. (**c**) Stacked-IMDB-GRU. (**d**) IMDB-GRU.

The comparison of RConC and NC of each layer of MNIST and IMDB models is shown in Figure 7 and Figure 8. First, we find that the RConC of the input layers of Stacked MNIST-LSTM, MNIST-LSTM, Stacked MNIST-GRU, and MNIST-GRU are smaller than the NC for the same inputs under different thresholds, which is consistent with our findings in Figure 6. However, for LSTM or GRU layers or other Dense layers, RConC is not necessarily smaller than NC. The contribution of the same neuron to the next layer is greatly affected by the weights. RNNCon takes this into account and combines the neuron output with the weight. In the trained model, for the same neuron, the weight matrix of its connections to the next layer is a constant matrix. We normalize the output of the neuron and the product of neuron output and weight matrix to 0 and 1 and compare the RConC and NC under the same threshold. We analyze that the reason why the RConC of the input layer is less than NC is that the weight matrix of the input layer connected to the next layer retains important features. For unimportant features, the weight values are set to be no greater than 1 or negative weakly retained or discarded. Under the same threshold, for the same neuron, if neuron coverage [13] is used, the output of the neuron is greater than the threshold, but if RNNCon is used, the RConC is smaller than the threshold. The features that can be processed by the non-input layer are relatively more important, so RConC > NC will occur. Therefore, RNNCon is used to assist the Neuron coverage, rather than replace it.

Secondly, we find that for the models in Figure 7, the RConC of LSTM or GRU and Dense of the last two layers are always much greater than that of NC. This shows that the weights of the last two layers have a great impact. Compared with neuron coverage, RNNCon is more accurate in measuring the transmission of information in the model due to the combination of weights. Although the inactivated contributions are far less than that of neurons, it can generate more effective adversarial samples and reduce the cost.

Thirdly, “All” in Figure 7 and Figure 8 indicates the coverage of the entire model, including all layers except the output layer. We find that except Stacked IMDB-LSTM and IMDB-LSTM, when the threshold is 0.5, RConC in “All” > NC in “All”. Other models have RConC in “All” > NC in “All” under all thresholds. For these models with high accuracy, RNNCon shrinks the search space for generating adversarial samples and measures the internal mechanism of the model more accurately. For Stacked IMDB-LSTM, IMDB-LSTM, Stacked IMDB-GRU, and IMDB-GRU, we consider that the difference between the internal structures of LSTM and GRU units leads to RConC > NC. There are three gates in the LSTM unit, namely, forget gate, input gate, and output gate. There are two doors in the GRU unit, the update door, and the reset door. The update gate is similar to the combination of LSTM forget and input gates. It determines which information to discard and which new information to add. We analyze that when the update gate discards information, the weight is small, resulting in a smaller contribution value, making RConC > NC.

Finally, we also find that RConC decreases with the increase of the number of layers under all thresholds. We analyze this because important features are constantly being accurately located.

In general, RNNCon can clearly show how the amount of information in the input data changes at each layer of the model and to some extent can reflect the difference between neural networks of the same architecture trained on different data sets. Compared with neuron coverage, RNNCon has a more accurate presentation of the process of information processing, for example, in the MNIST-LSTM model in Figure 7, the coverage of neurons is 99.22%, 53.78%, and 90.00% in the Input layer, LSTM128 layer, and Dense32 layer, respectively, while the contribution coverage is 83.25%, 96.88%, and 90%, respectively. Because the LSTM128 layer has the function of “memory” and information accumulation, the expectation is that both the neuron coverage and the contribution coverage of LSTM128 are larger than the coverage of the input layer, but this does not match our actual results, indicating that the neuron coverage metric loses a part of the logic inside the RNNs. Furthermore, it shows that the coverage of RNNCon is stronger than the neuron coverage in RNNs.

It should be noted that during the coverage statistics of the model in Figure 7, we take each one-dimensional feature of the input image as the neuron output of the input layer and then multiply it with the weight matrix to obtain the contribution coverage of the input layer. However, for Figure 8, we exclude the Embedding layer in the model. This is because the contribution extraction and coverage calculation of the Embedding layer are time-consuming and meaningless. Since the neuron coverage metric is oriented to CNNs, we use the elements of the hidden state vector as neurons in our implementation.

Answer to RQ1: RNNCon has better coverage capacity than neuron coverage. We suggest using RNNCon to guide the generation of test inputs rather than using alone or using neuron coverage. RNNCon can more accurately measure the transmission of information in the model.

### 5.3. Effectiveness of RNNCon-Test (RQ2)

**Figure 9:**
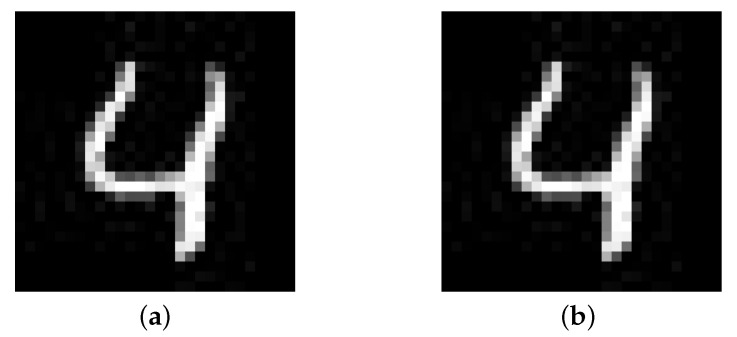
The Adversarial sample generated by RNNCon-Test cannot be distinguished from the original seed by human eyes. (**a**) Original seed, result:4. (**b**) Adversarial sample, result:3.

In order to conduct a comprehensive evaluation, we provide the same 500 seeds to Stacked MNIST-LSTM, MNIST-LSTM, Stacked MNIST-GRU, and MNIST-GRU to generate adversarial samples. Figure 9 shows a sample extracted from the experimental data.

It can be found that human eyes cannot distinguish between the original seed and the adversarial sample, but the model MNIST-GRU correctly predicts the value of the original seed to be 4, and the value of adversarial sample to be 3. Table 2 summarizes the results of generating adversarial samples under the guidance of RNNCon.

Where #Layer represents the layer where the inactivated contributions we want to activate is located, Random means that we randomly select the inactived contribution to activate at each layer. Constraint indicates that we generate test inputs by simulating shot pollution. We generate one test input for each seed. #Adv.Inputs represents the number of adversarial samples produced by 500 seeds. #Avg.Perturb(L2norm) represents the mean value of the perturbations superimposed on the original seeds when generating the adversarial inputs. #Adv.Rate indicates the percentage of adversarial samples produced by 500 seeds.

We can find that in all MNIST models, #Adv.Rate and #Avg.Perturb of adversarial samples generated by activating inactivated contributions are greater than those generated only by adding Constraint. However, adding constraints produces a low #Avg.Perturb of the adversarial sample. This indicates that when generating the adversarial samples, we need to balance the perturbation and the generation rate of the adversarial samples, i.e., if we want a high generation rate of the adversarial samples, we have to tolerate the problem of generating a large perturbation. In the models Stacked-MNIST-LSTM, MNIST-LSTM, and MNIST-GRU, the #Adv.Rate generated for selecting inactivated neurons in a single layer is higher than the #Adv.Rate generated by randomly selecting in all layers, which indicates that this single layer of the model is more vulnerable to attacks compared to other layers. This indicates that RNNCon-Test can effectively activate the unactivated contributions and is effective in generating adversarial samples.

A comparison of the effectiveness of RNNCon-Test with RNN-Test on the model MNIST-LSTM, given 500 original seeds, is shown in Table 3. It can be found that the adversarial sample generation rate Adv.Rate of RNNCon-Test is 84.4%, while that of RNN-Test is 69.6%, with a difference of 14.8%, for the same number of test inputs generated. The perturbation applied by RNNCon-Test is 0.139, which is much smaller than the 1.740 of RNN-Test. It is well known that it is better to have more adversarial samples generated with less perturbation. So, RNNCon-Test is more effective than RNN-Test.

Table 4 shows a performance comparison between RNNCon-Test and RNN-Test [28]. Our framework RNNCon-Test can reduce the performance of the model more than RNN-Test. For example, in the model MNIST-GRU, the accuracy of RNNCon-Test was 10.80%, which was 9.2% lower than that of RNN-Test. The success rate of generating adversarial samples Adv.Rate is 89.20%, which is 9.89% higher than that of RNN-Test.

**Figure 10:**
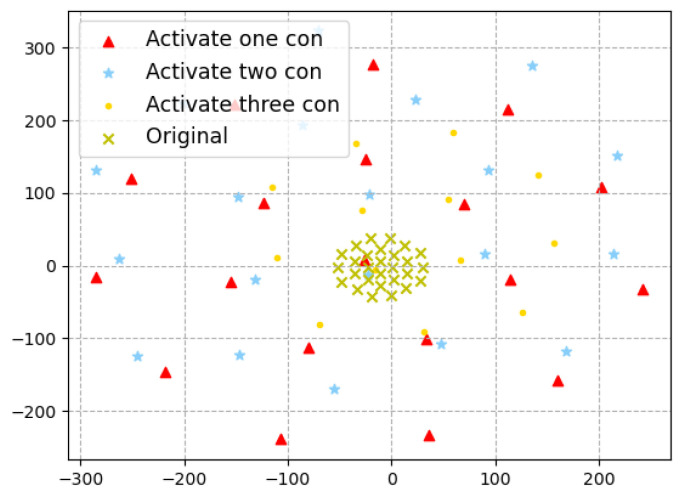
TSNE transformations of perturbations obtained by activating different numbers of contributions of RNNCon-Test in model MNIST-GRU for one same test input.

The high-dimensional reduction technique TSNE [47] transformation of the perturbations generated by activating different number of inactivatedinactive contributions for one same test input, as shown in Figure 10. Although the distribution of perturbations produced by activating different amounts of contributions is uniform, activating three contributions is closer to the original distribution than activating one or two contributions. It indicates that the adversarial space generated by activating three contributions will be more limited than that generated by activating fewer contributions, so the diversity of the generated adversarial sample set will be relatively small. So, when we want to generate adversarial samples, it is not always the case that it is better to have more contributions activated at the same time. This also indicates that RNNCon-Test can effectively activate the inactivated contributions to generate adversarial samples.

**Figure 11:**
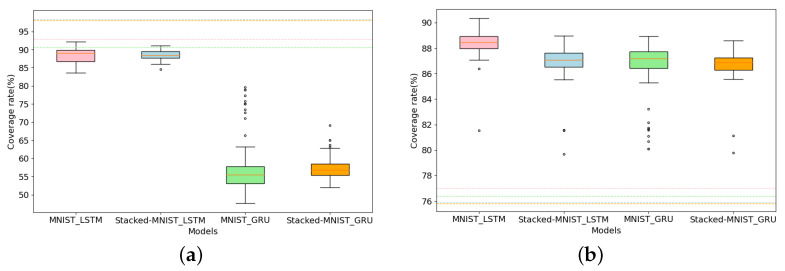
Compare the coverage rate of different RNN models before and after adding adversarial samples to the same MNIST seed dataset. The horizontal dotted lines represent the average coverage rate of the model corresponding to the same color when no adversarial samples are fed. The boxes represent the coverage value ranges of the model after being fed with adversarial samples. (**a**) MNIST-NC. (**b**) MNIST-RConC.

For different models, the comparison before and after adding the generated adversarial samples to the same MNIST seed set to calculate the coverage is shown in Figure 11. Figure 11a,b generate adversarial samples by activating inactivated neurons and contributions, respectively. We find that the NC is much higher than RConC before the model is fed with adversarial samples. After feeding the adversarial samples generated under the guidance of the neuron coverage metric and RNNCon, the value range of NC is smaller than the original average coverage in the four models of Stacked MNIST-LSTM, MNIST-LSTM, Stacked MNIST-GRU, and MNIST-GRU, and the value range of RNNCon is much higher than the original average coverage. Obviously, the adversarial samples generated by activating neurons should increase the coverage rate of neurons, but it actually decreases, which indicates that the neuron coverage metric is not applicable to Stacked RNNs. This further illustrates the effectiveness of RNNCon for testing RNNs.

**Figure 12:**
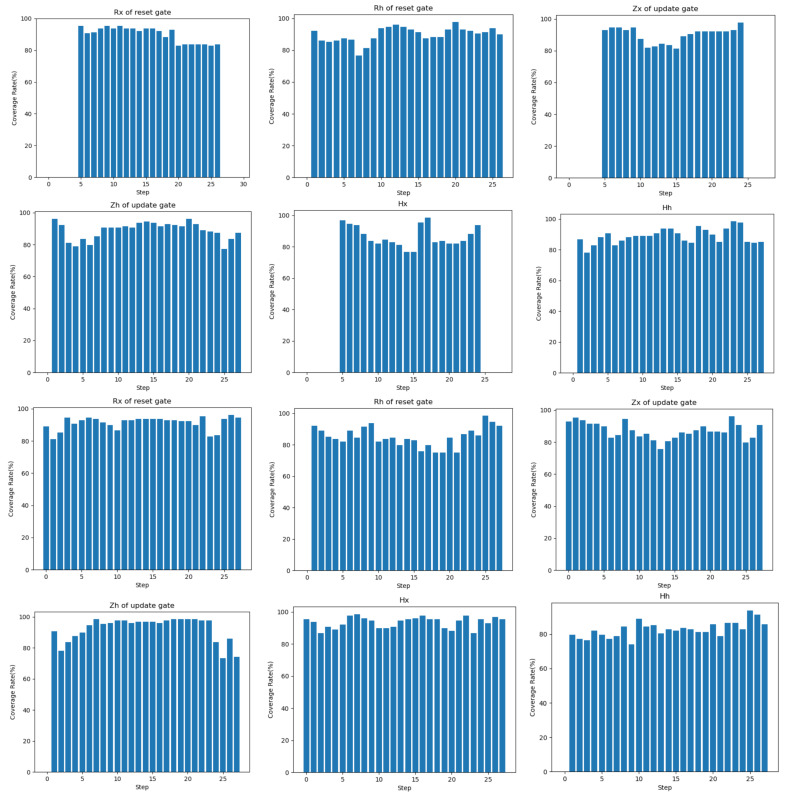
The coverage of each gate at each time step after feeding the original seed and adversarial sample to the MNIST-GRU model. The first two rows represent the coverage rate of the original seed. The last two lines represent the coverage rate obtained by feeding the adversarial sample generated by the same original seed. “Rx of reset gate” and “Rh of reset gate” are the coverage of reset gate. “Zx of update gate” and “Zh of update gate” are the coverage of the update gate. “Hx” and “Hh” are the coverage of hidden states.

We visualize the coverage of reset and update gates in the GRU at each time step after the MNIST-GRU model is fed the original seed and the adversarial sample, as shown in Figure 12. “Rx of reset gate” and “Rh of reset gate” represent the coverage of the current input and the information left by the previous time step for each time step processed by the update gate.. The update gate determines how much information from the past needs to be forgotten. We can see that the coverage of time steps 1–4 and 26–28 is zero when the original seed of the model is fed and the update gate processes the current input *x*. Considering that the input for each time step processing is each column of pixels of the image, the update gate pays more attention to the intermediate time steps because the digital part of MNIST data is located in the center of the image. As can be seen, the update gate also focuses more on the intermediate time step. When we feed the adversarial sample, we find that coverage increases at time steps 1–4 and 26–28. So, we analyze that these perturbations that we generate are added to the original seed, strengthening the features of some parts of the image that are not particularly important to the prediction process.

Answer to RQ2: The contribution coverage metric RNNCon as a guide generated adversarial samples is superior to RNN-Test [28] and neuron coverage [13]. Covering only neurons is not applicable to Stacked RNNs. Compared with RNN-Test, RNNCon-Test can significantly reduce the performance of the model and has a stronger ability to generate adversarial inputs, which is 9.89% higher. Even a small amount of contribution can be activated to generate adversarial inputs in a high proportion. RNNCon-Test has the potential to become more effective because of divergent perturbations.

### 5.4. Quality of Adversarial Input (RQ3)

We assess the quality of the adversarial samples generated by RNNCon-Test by IS [39] and FID [40] and L1-distance. The L1-distance measures the similarity between the original seed and the adversarial sample, and the smaller the L1-distance the better in order to make the perturbation undetectable to the human eye. IS measures the degree of similarity between two distributions, and to improve the clarity and diversity of the dataset, then the larger the IS, the better. Because IS does not take into account the effect of real-world data, the FID can be used to determine whether the distribution of the measured objects is similar to the true distribution. Therefore, the smaller the FID, the better. In summary, we prefer the dataset with the smallest L1-distance for retraining. When L1-distance is indistinguishable, we choose the smallest FID. when both L1-distance and FID are indistinguishable, we choose the dataset with the largest IS.

Table 5 shows the L1-distance, IS, FID and the accuracy of the model after retraining with different datasets, where the dataset is the set of data formed by adding the corresponding generated adversarial samples to the training set. Table 5 shows the L1-distance, IS, FID and the accuracy of the model after retraining with different datasets, where the dataset is the set of data formed by adding the corresponding generated adversarial samples to the training set. For the supervised task, we use the labels of the original seeds that generate the adversarial samples as the labels of the adversarial samples.

First, from Table 5 we can find that, except the MNIST-GRU model, the model retrained with the smallest L1-distance training set has the highest accuracy. For example, in the model Stacked-MNIST-LSTM, when the L1-distance is the smallest, 2821, the accuracy after retraining is the highest, 99.12. The training set with the smallest FID obtained in MNIST-LSTM and MNIST-GRU has a higher accuracy of the retrained model. Only the training set with the largest IS in the MNIST-LSTM model has a higher accuracy after the model is retrained. These results are consistent with our expectations, so we suggest that when selecting a dataset for the retraining process, the one with the smallest L1-distance is preferred, followed by the one with the smallest FID, and only the one with the largest IS is selected last.

We use Accuracy, Precision, Recall, F1 Score, and AUC, which are deep learning model performance evaluation metrics to measure the performance of the model. The performance improvement after retraining is shown in Table 6. We can find that in the four models Stacked MNIST-LSTM, MNIST-LSTM, Stacked MNIST-GRU and MNIST-GRU, the accuracies of the same test set have been improved after retraining. If the accuracies are high before retraining, it can be improved by 0.45% at most. The precision can be improved by 0.47% at most. The recall rate can be increased by 0.46% at most. The F1 score can be increased by 0.44 at most. Area Under Curve (AUC) is not improved much.

**Figure 13:**
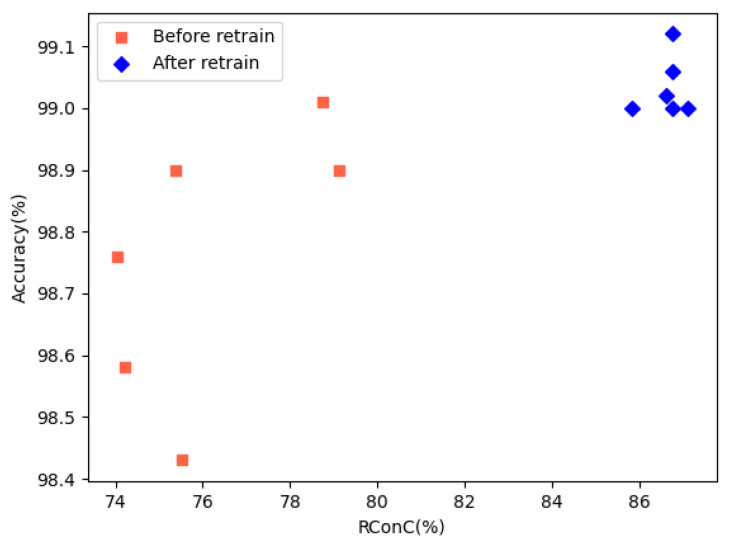
Comparison of RConC and accuracy before and after retraining.

The comparison of RConC and accuracy before and after retraining is shown in Figure 13. We can see that after retraining, the RConC and accuracy have been effectively improved. We find that the RNNCon is not related to the accuracy after retraining, which is consistent with Harel-Canada et al.’s opinion [35].

**Figure 14:**
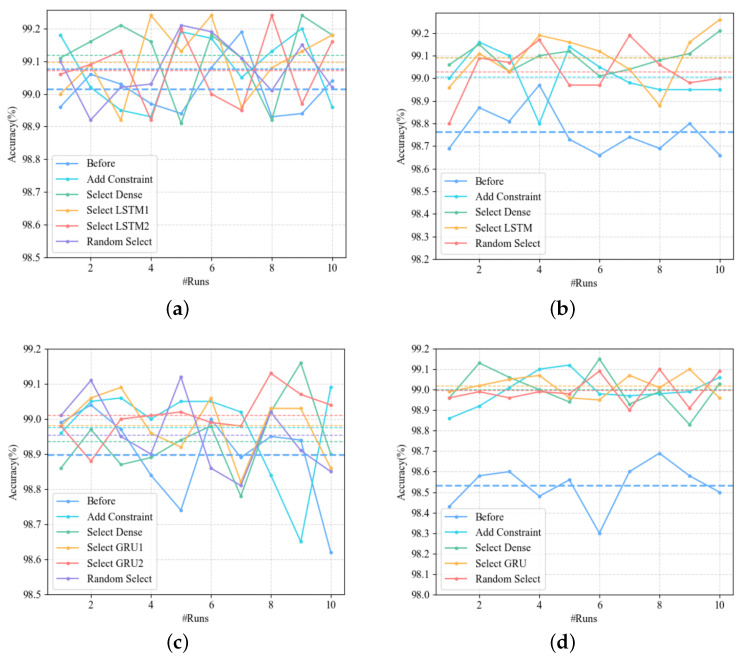
Accuracy comparison before and after the retraining of the corresponding model with the addition of the adversarial samples to the training set, a total of 10 runs. “Before” indicates accuracy without retraining. “Add Constraint” means to generate adversarial samples by adding small black rectangles to the original seed to simulate camera lens stains. “Select Dense”, “Select LSTM”, “Select GRU” and “Random select” refer to the selection of the layer where the inactive contributions can be selected when the adversarial samples are generated, which are respectively Dense layer, LSTM layer, GRU layer and all layers except the output layer. The dotted line is the average accuracy of 10 runs, and the color is consistent with the corresponding polyline. (**a**) Stacked-MNIST-LSTM. (**b**) MNIST-LSTM. (**c**) Stacked-MNIST-GRU. (**d**) MNIST-GRU.

The accuracy comparison before and after retraining is shown in Figure 14. Due to the uncertainty of the models, we retrain each model 10 times, and the dotted line is the average accuracy. The accuracy of the four models in Figure 14 has been significantly improved after retraining. However, the selection of the layer in which the inactivated contribution is located has only a small effect on improving the accuracy of the model.

Answer to RQ3: We filter the adversarial inputs by minimum L1-distance, minimum FID, and maximum IS, making them natural and close to the real world. After automatically marking these adversarial samples with the original labels, adding them to the training set to retrain the model can improve the performance of the model.

## 6. Threats to Validity

First of all, for the MNIST dataset, we set the threshold value to 0.25 when conducting experiments. When we increase the threshold value, the coverage becomes lower, and when we reduce the threshold value, the coverage increases. For the IMDB dataset, we set the threshold value to 0.5, and the method of threshold selection remains a legacy issue. Second, when selecting the inactivated contributions in a certain layer, we adopt a random selection method, which brings a certain threat of uncertainty to our experimental results. The prediction results of the DL systems have a strong uncertainty. When we retrain, we load the original weight information. On this basis, we continue to run for 10 rounds to avoid this uncertainty to some extent.

## 7. Discussion

In this section, we discuss the limitations and potential problems of our work.

Correctness is a measure of the probability that a DL system is “correct”. The correctness of a model is the probability that the model’s predicted label for an input is equal to its true label. Robustness is defined by the IEEE standard glossary of software engineering terminology as the degree to which a system or component can function correctly under invalid inputs or stressful environmental conditions. (1) Let *S* be a DL system. Let E(S) be the correctness of *S*, σ(S) be the perturbations of *S* on any machine learning components e.g., data, learning program, or framework. The robustness of the DL system is a measure of the difference between E(S) and E(σ(S)) [8]. We train model *s* and model σ(s) by choosing the same MNIST-GRU network architecture, MNIST dataset, and different epoch values so that the model σ(s) was over-fitted to simply simulate that the system being disturbed, and then obtained robustness by calculating the difference of the correctness between the models *s* and σ(s). We generate 500 test inputs for model *s*, automatically label them with the same labels as the original seeds, and add them to the training set, then retrain the model. We find significant improvement in robustness. (2) We evaluate RNNCon on small Stacked RNNs. In the real world, the types of RNNs are large, and the datasets processed include not only text types but also sequence data such as speech. Our next work will evaluate RNNCon and RNNcon-Test on more and larger stacked RNNS, as well as the applicability of RNNCon to CNNs.

## 8. Related Work

Pei et al. [13] defined the neuron coverage metric for the first time, and proposed the concept of neuron coverage rate (i.e., the ratio of the number of neurons whose output values are greater than the specified threshold to the total number of neurons) to measure the test adequacy of DNNs. They generate test inputs by jointly maximizing the neuron coverage rate and the differential behavior of the model. The generated test inputs can not only be extended to the training dataset to retrain the model to improve the accuracy and robustness of the model but also detect possible data pollution attacks in the training dataset. Although the neuron coverage metric is oriented to CNNs and cannot be accurately applied directly to RNNs, inspired by it, we tried to find out the real neurons in RNNs by analyzing the internal structure of RNNs and its variants LSTMs and GRUS.

With the introduction of the neuron coverage metric, many studies have extended it with finer granularity and proposed many related test coverage metrics. Sun et al. [16] formalized several DNN coverage metrics that had been proposed at that time, and provided them with a unified test input generation algorithm to maximize the coverage rate, to a certain extent, eased the trend of the coverage criteria towards customization.

Zhou et al. [19] pointed out that using only the output of a neuron to determine the activation state of a neuron is incomplete since the prediction results of the DNNs were determined by the outputs of the neurons and the weights it emit. Therefore, Zhou et al. [19] defined the concept of the term “Contribution”, that is, the combination of a neuron output and the weight it emits and then proposed a contribution coverage metric for CNNs, which was used to guide the generation of tests and measure the adequacy of testing. Inspired by Zhou et al.’s study, we believe that the weights emitted by neurons in RNNs are equally important. However, unlike the network structure of CNNs, the neurons of the layers of RNNs are connected. The contributions oriented to CNNs are difficult to locate accurately until the neurons and weights in RNNs are analyzed. Therefore, we redefine the contribution of RNNs. Unlike the studies by Pei et al. and Zhou et al., our study is on RNNs and the redefined contributions in RNNs are taken as the minimum unit of test coverage. Similar to Sun et al.’s study of maximizing neuron coverage we also generated test inputs by maximizing contribution coverage, but we further calculated the ratio of adversarial samples in the generated test inputs. This is because the generated test inputs may be invalid and do not reveal the flaws of the model as much as the adversarial samples.

Guo et al. [22] proposed a differential fuzzing framework. It worked by constantly mutating the input subtly in order to maximize the neuron coverage and prediction difference between the original and mutated inputs without manually labeling or cross-referencing test oracles from other DL systems of the same function. Compared with Pei et al.’s study [13], it can achieve higher coverage and obtain more abnormal behavior. The way of generating adversarial samples in our study is similar to that of Guo et al. The difference is that we are oriented to RNNs with memory functions and maximize contribution coverage rather than neuron coverage.

Harel-Canada et al. [35] and Abrecht et al. [32] argue that the correlation between high neuron coverage and the quality of DNNs is elusive. Therefore, we measure the contribution coverage along with the commonly used performance metrics to examine the variation in the performance of DNNs.

The researches of Du et al. [30], Huang et al. [29], and Guo et al. [28] are rare works conducted toward RNNs. Du et al. model an RNN model as an abstract state-transition system to characterize its internal behavior, and coverage tests based on that system. However, as the number of layers of the neural network increases, the process is time-consuming and labor-intensive. In contrast, our work is performed by unfolding an RNN along time steps by a technique called computational graph unfolding, which matches the actual computing process and thus saves costs. The work of Huang et al. and Guo et al. is mentioned in “Limitations of existing coverage metrics”. Our study differs from theirs in that we take the contribution of the underlying recurrent cell as the minimum test unit instead of the entire recurrent cell, and our study can explore the internal decision mechanism of RNNs more deeply.

## 9. Conclusions

DL systems are widely used in various security fields, which puts forward higher requirements for the robustness of DL systems. The coverage metrics of existing DL systems are oriented to CNNs, and limited testing efforts are oriented to Stacked RNNs. In this paper, we propose a contribution coverage metric RNNCon that applies Stacked RNNs.

RNNCon divides the contribution extraction in Stacked RNNs into two categories. One is that at a single time step, the contribution is expressed as the combination of the output of a neuron and the weight it emits. The second category is over multiple time steps, where the contribution is expressed as the combination of the output of a hidden neuron at the previous time step and the weight it emits to connect the same hidden neuron at the next time step.

Experiments with different datasets and on different models show that for a single RNN layer, RNNCon can cover deeper internal decision mechanisms and has stronger coverage compared to neuronal coverage.

Our further designed and implemented prototype framework RNNCon-Tes can efficiently activate the inactivated contributions to generate adversarial samples with a success rate of 84.4% in MNIST-LSTM, which is 14.8% higher than the current state-of-the-art study RNN-Test, and generates a small perturbation of 0.139, compared to the perturbation of RNN-Test is reduced by 1.601. We add the generated adversarial samples to the training data set, and retrain the model by sequentially screening the data sets with small L1-distance, small FID, and large IS, which can effectively improve the performance of the model, and improve the accuracy rate up to 0.45%, precision rate up to 0.47%, recall rate up to 0.46% and F1 score up to 0.44 when the performance of the model is already at a high level. In the future, we plan to evaluate the applicability of RNNCon to large Stacked RNNs or CNNs. We also plan to investigate RNNCon-based adversarial defense methods.

## Figures and Tables

**Table 1:** Summary of datasets and RNN models.

Dataset	No. of Classes	Model Name	Main Architecture	Accuracy (%)		#Parameter	#Neurons	#Cons
				Reported	Ours			
MNIST	10	Stacked-MNIST-LSTM	Two-layer LSTM	98.70	99.01	216,426	3754	58,520
		MNIST-LSTM	One-layer LSTM	96.88	98.76	84,842	170	29,848
		Stacked-MNIST-GRU	Two-layer GRU	-	98.90	164,202	3754	44,184
		MNIST-GRU	One-later GRU	96.50	98.53	65,130	170	22,680
IMDB	2	Stacked-IMDB-LSTM	Two-layer LSTM	-	84.68	1,818,177	64,161	66,592
		IMDB-LSTM	One-layer LSTM	86.20	87.24	1,686,593	161	33,824
		Stacked-IMDB-GRU	Two-layer GRU	-	86.21	1,765,441	64,161	50,208
		IMDB-GRU	One-layer GRU	-	86.59	1,666,369	161	25,632

**Table 2:** Generate adversarial samples guided by RNNCon.

Model Name	#Layer	#Seeds	#Adv.Inputs	Avg.Perturb (L2 Norm)	Adv.Rate (%)
Stacked-MNIST-LSTM	Random	500	428	0.334	85.60
	LSTM1		436	0.321	87.20
	LSTM2		424	0.210	84.80
	Dense		358	0.061	71.60
	Constraint		355	0.002	71.00
MNIST-LSTM	Random	500	422	0.139	84.40
	LSTM		443	0.170	88.60
	Dense		402	0.113	84.40
	Constraint		361	0.029	72.20
Stacked-MNIST-GRU	Random	500	444	0.282	88.80
	GRU1		435	0.266	87.00
	GRU2		441	0.231	88.20
	Dense		418	0.044	83.60
	Constraint		351	0.029	70.20
MNIST-GRU	Random	500	422	0.191	84.40
	GRU		427	0.208	85.40
	Dense		446	0.127	89.20
	Constraint		396	0.029	79.20

**Table 3:** Effectiveness of RNNCon-Test compared to RNN-Test [28] on MNIST-LSTM model over 500 original seeds.

Methodology	#Seeds	#Adv.Inputs	Avg.Perturb (L2 Norm)	Adv.Rate (%)
RNN-Test	500	348	1.740	69.6
RNNCon-Test	500	422	0.139	84.4

**Table 4:** Performance comparison with RNN-test [28].

Model	Performance	Original	RNN-Test	RNNCon
MNIST-GRU	Accuracy (%)	98.53	20.00	10.80
	Adv.Rate (%)	-	79.31	89.20

**Table 5:** Choose natural, real-world datasets for retraining.

Model	#Layer	Coverage Rate (RConC) (%)	L1-Distance	IS	FID	Acc.Retrain
Stacked-MNIST-LSTM	Random	86.85	3867	2.299	222.348	99.07
	LSTM1	86.79	3837	2.141	221.004	99.10
	LSTM2	86.88	3979	1.682	222.055	99.07
	Dense	86.11	**2821**	2.093	224.557	**99.12**
	Constraint	85.76	2315	1.833	224.559	99.08
MNIST-LSTM	Random	87.54	3465	2.176	223.572	99.03
	LSTM	87.91	3547	2.025	223.993	99.09
	Dense	86.43	1267	2.179	222.467	99.09
	Constraint	85.36	2324	1.866	224.981	99.01
Stacked-MNIST-GRU	Random	86.24	3710	2.565	222.750	98.94
	GRU1	86.27	3697	2.382	222.901	98.98
	GRU2	86.32	3770	1.946	222.566	99.01
	Dense	85.69	2632	2.277	222.956	98.94
	Constraint	85.35	2256	1.892	224.592	99.02
MNIST-GRU	Random	85.91	3433	2.225	221.678	99.00
	GRU	86.20	3508	2.031	221.536	99.02
	Dense	85.09	3013	2.222	222.215	99.00
	Constraint	84.17	2281	1.871	223.045	99.00

**Table 6:** Improved performance after retraining.

Model	Before					After				
	Acc. (%)	Precision (%)	Recall (%)	F1	AUC	Acc. (%)	Precision (%)	Recall (%)	F1	AUC
Stacked-MNIST-LSTM	98.72	98.74	98.70	98.72	99.99	99.17	99.17	99.16	99.16	99.99
MNIST-LSTM	98.93	98.93	98.91	98.92	99.98	99.11	99.10	99.10	99.10	99.99
Stacked-MNIST-GRU	98.79	98.81	98.77	98.78	99.98	99.01	99.01	99.00	99.00	99.99
MNIST-GRU	98.68	98.67	98.67	98.66	99.99	99.04	99.04	99.03	99.03	99.99

## Data Availability

The data presented in this research are available on request from the corresponding author.

## References

[1] Collobert R., Weston J. A unified architecture for natural language processing: Deep neural networks with multitask learning. Proceedings of the 25th International Conference on Machine Learning.

[2] Mikolov T., Chen K., Corrado G.S., Dean J. Efficient Estimation of Word Representations in Vector Space. Proceedings of the International Conference on Learning Representations.

[3] Krizhevsky A., Sutskever I., Hinton G.E. (2017). ImageNet Classification with Deep Convolutional Neural Networks. Commun. ACM.

[4] Hinton G., Deng L., Yu D., Dahl G.E., Mohamed A.R., Jaitly N., Senior A., Vanhoucke V., Nguyen P., Sainath T.N. (2012). Deep neural networks for acoustic modeling in speech recognition: The shared views of four research groups. IEEE Signal Process. Mag..

[5] Chen B., Jiang J., Wang X., Wan P., Wang J., Long M. (2022). Debiased Self-Training for Semi-Supervised Learning. arXiv.

[6] Bojarski M., Testa D.D., Dworakowski D., Firner B., Flepp B., Goyal P., Jackel L.D., Monfort M., Muller U., Zhang J. (2016). End to End Learning for Self-Driving Cars. arXiv.

[7] Djolonga J., Yung J., Tschannen M., Romijnders R., Beyer L., Kolesnikov A., Puigcerver J., Minderer M., D’Amour A., Moldovan D. On robustness and transferability of convolutional neural networks. Proceedings of the IEEE/CVF Conference on Computer Vision and Pattern Recognition.

[8] Zhang J.M., Harman M., Ma L., Liu Y. (2022). Machine learning testing: Survey, landscapes and horizons. IEEE Trans. Softw. Eng..

[9] Lipton Z.C. (2016). The Mythos of Model Interpretability. arXiv.

[10] Molnar C., Casalicchio G., Bischl B. (2020). Interpretable machine learning—A brief history, state-of-the-art and challenges. Proceedings of the Joint European Conference on Machine Learning and Knowledge Discovery in Databases.

[11] Adebayo J., Gilmer J., Muelly M., Goodfellow I.J., Hardt M., Kim B. (2018). Sanity Checks for Saliency Maps. arXiv.

[12] Berend D. (2021). Distribution Awareness for AI System Testing. arXiv.

[13] Pei K., Cao Y., Yang J., Jana S. Deepxplore: Automated whitebox testing of deep learning systems. Proceedings of the 26th Symposium on Operating Systems Principles.

[14] Ma L., Juefei-Xu F., Zhang F., Sun J., Xue M., Li B., Chen C., Su T., Li L., Liu Y. Deepgauge: Multi-granularity testing criteria for deep learning systems. Proceedings of the 33rd ACM/IEEE International Conference on Automated Software Engineering.

[15] Ma L., Juefei-Xu F., Xue M., Li B., Li L., Liu Y., Zhao J. (2019). Deepct: Tomographic combinatorial testing for deep learning systems. Proceedings of the 2019 IEEE 26th International Conference on Software Analysis, Evolution and Reengineering (SANER).

[16] Sun Y., Wu M., Ruan W., Huang X., Kwiatkowska M., Kroening D. (2018). Concolic Testing for Deep Neural Networks. arXiv.

[17] Sun Y., Huang X., Kroening D., Sharp J., Hill M., Ashmore R. (2018). Testing deep neural networks. arXiv.

[18] Sun Y., Huang X., Kroening D., Sharp J., Hill M., Ashmore R. (2019). Structural test coverage criteria for deep neural networks. ACM Trans. Embed. Comput. Syst..

[19] Zhou Z., Dou W., Liu J., Zhang C., Wei J., Ye D. DeepCon: Contribution Coverage Testing for Deep Learning Systems. Proceedings of the 2021 IEEE International Conference on Software Analysis, Evolution and Reengineering (SANER).

[20] Xie X., Ma L., Juefei-Xu F., Xue M., Chen H., Liu Y., Zhao J., Li B., Yin J., See S. (2019). DeepHunter: A Coverage-Guided Fuzz Testing Framework for Deep Neural Networks. Proceedings of the 28th ACM SIGSOFT International Symposium on Software Testing and Analysis.

[21] Tian Y., Pei K., Jana S., Ray B. Deeptest: Automated testing of deep-neural-network-driven autonomous cars. Proceedings of the 40th International Conference on Software Engineering.

[22] Guo J., Jiang Y., Zhao Y., Chen Q., Sun J. (2018). DLFuzz: Differential Fuzzing Testing of Deep Learning Systems. Proceedings of the 2018 26th ACM Joint Meeting on European Software Engineering Conference and Symposium on the Foundations of Software Engineering (ESEC/FSE 2018).

[23] Chen Y. (2015). Convolutional Neural Network for Sentence Classification. Master’s Thesis.

[24] Mikolov T., Karafiát M., Burget L., Cernockỳ J., Khudanpur S. Recurrent neural network based language model. Proceedings of the INTERSPEECH 2010—11th Annual Conference of the International Speech Communication Association, Makuhari.

[25] Egmont-Petersen M., de Ridder D., Handels H. (2002). Image processing with neural networks—A review. Pattern Recognit..

[26] Sun Y., Zheng W., Ren Z. (2022). Application of convolutional neural network in image processing. Proceedings of the International Conference on Multi-modal Information Analytics.

[27] Medhat W., Hassan A., Korashy H. (2014). Sentiment analysis algorithms and applications: A survey. Ain Shams Eng. J..

[28] Guo J., Zhang Q., Zhao Y., Shi H., Jiang Y., Sun J. (2021). Rnn-test: Towards adversarial testing for recurrent neural network systems. IEEE Trans. Softw. Eng..

[29] Huang W., Sun Y., Zhao X., Sharp J., Ruan W., Meng J., Huang X. (2021). Coverage-guided testing for recurrent neural networks. IEEE Trans. Reliab..

[30] Du X., Xie X., Li Y., Ma L., Liu Y., Zhao J. (2019). DeepStellar: Model-Based Quantitative Analysis of Stateful Deep Learning Systems. Proceedings of the 2019 27th ACM Joint Meeting on European Software Engineering Conference and Symposium on the Foundations of Software Engineering (ESEC/FSE 2019).

[31] Papernot N., Mcdaniel P., Swami A., Harang R. Crafting Adversarial Input Sequences for Recurrent Neural Networks. Proceedings of the MILCOM 2016—2016 IEEE Military Communications Conference.

[32] Abrecht S., Akila M., Gannamaneni S.S., Groh K., Heinzemann C., Houben S., Woehrle M. (2020). Revisiting Neuron Coverage and Its Application to Test Generation. Proceedings 39, Computer Safety, Reliability, and Security. SAFECOMP 2020 Workshops: DECSoS 2020, DepDevOps 2020, USDAI 2020, and WAISE 2020, Lisbon, Portugal, 15 September 2020.

[33] Pascanu R., Gulcehre C., Cho K., Bengio Y. (2013). How to construct deep recurrent neural networks. arXiv.

[34] Hermans M., Schrauwen B., Burges C., Bottou L., Welling M., Ghahramani Z., Weinberger K. (2013). Training and Analysing Deep Recurrent Neural Networks. Advances in Neural Information Processing Systems.

[35] Harel-Canada F., Wang L., Gulzar M.A., Gu Q., Kim M. (2020). Is Neuron Coverage a Meaningful Measure for Testing Deep Neural Networks?. Proceedings of the 28th ACM Joint Meeting on European Software Engineering Conference and Symposium on the Foundations of Software Engineering (ESEC/FSE 2020).

[36] Cho K., van Merriënboer B., Gulcehre C., Bahdanau D., Bougares F., Schwenk H., Bengio Y. (2014). Learning Phrase Representations using RNN Encoder–Decoder for Statistical Machine Translation. Proceedings of the 2014 Conference on Empirical Methods in Natural Language Processing (EMNLP).

[37] Hochreiter S., Schmidhuber J. (1997). Long Short-term Memory. Neural Comput..

[38] Chung J., Gulcehre C., Cho K., Bengio Y. (2014). Empirical Evaluation of Gated Recurrent Neural Networks on Sequence Modeling. arXiv.

[39] Salimans T., Goodfellow I., Zaremba W., Cheung V., Radford A., Chen X. (2016). Improved techniques for training gans. Adv. Neural Inf. Process. Syst..

[40] Heusel M., Ramsauer H., Unterthiner T., Nessler B., Hochreiter S. (2017). Gans trained by a two time-scale update rule converge to a local nash equilibrium. Adv. Neural Inf. Process. Syst..

[41] Elman J.L. (1990). Finding structure in time. Cogn. Sci..

[42] Wei J., Zou K. (2019). Eda: Easy data augmentation techniques for boosting performance on text classification tasks. arXiv.

[43] LeCun Y., Cortes C., Burges C.J.C. (2010). MNIST Handwritten Digit Database. http://yann.lecun.com/exdb/mnist/.

[44] Maas A.L., Daly R.E., Pham P.T., Huang D., Ng A.Y., Potts C. (2011). Learning Word Vectors for Sentiment Analysis. Proceedings of the 49th Annual Meeting of the Association for Computational Linguistics: Human Language Technologies.

[45] Li Z., Ma X., Xu C., Cao C. Structural Coverage Criteria for Neural Networks Could Be Misleading. Proceedings of the 2019 IEEE/ACM 41st International Conference on Software Engineering: New Ideas and Emerging Results (ICSE-NIER).

[46] Dong Y., Zhang P., Wang J., Liu S., Sun J., Hao J., Wang X., Wang L., Dong J.S., Ting D. (2019). There is limited correlation between coverage and robustness for deep neural networks. arXiv.

[47] van der Maaten L., Hinton G. (2008). Visualizing Data using t-SNE. J. Mach. Learn. Res..

